# Predicting and identifying determinants of stunting among school-aged children in Ethiopia: a machine learning approach

**DOI:** 10.1186/s41043-026-01401-y

**Published:** 2026-07-12

**Authors:** Shibiru Jabessa Dugasa, Merga Abdissa Aga

**Affiliations:** 1 Department of Statistics, Wollega University, Nekemte, Oromia Ethiopia; 2 Department of Statistics, Salale University, Fiche, Oromia Ethiopia

**Keywords:** Stunting, School-aged children, Machine learning, Ethiopia, Classification

## Abstract

**Background:**

Childhood stunting remains a major public health concern, reflecting chronic undernutrition and long-term socioeconomic disadvantage. Among school-aged children, stunting is associated with impaired physical growth, reduced cognitive development, and poorer educational outcomes. Traditional statistical approaches, such as logistic regression, have been widely used to examine factors associated with stunting; however, their ability to capture complex and nonlinear relationships is limited. Machine learning (ML) methods provide a flexible alternative for modeling such relationships.

**Objective:**

This study aimed to model and classify stunting among school-aged children in Ethiopia using school- and household-level data, and to compare the performance of machine learning algorithms with multivariable logistic regression.

**Methods:**

A cross-sectional analysis was conducted using secondary data from Round 5 (2016–2017) of the Young Lives study in Ethiopia. Stunting was defined as a binary outcome based on World Health Organization height-for-age Z-score criteria. Several machine learning algorithms, including Random Forest, Support Vector Machine, and Gradient Boosting Machine, were implemented. Model performance was evaluated using accuracy, sensitivity, specificity, F1-score, and area under the receiver operating characteristic curve (AUC). Variable importance measures were used to identify predictors contributing to model performance. To avoid potential circularity, anthropometric variables closely related to the outcome (e.g., child weight) were excluded from the final models.

**Results:**

The Random Forest model demonstrated modestly improved performance compared with logistic regression and other machine learning methods, and its performance was evaluated using accuracy, sensitivity, specificity, AUC, and F1-score. Key predictors included school type, household wealth index, literacy-related indicators, and region of residence. Notable regional variation in stunting classification was observed, suggesting the influence of broader socioeconomic and environmental conditions.

**Conclusion:**

Machine learning models, particularly Random Forest, showed slightly better performance than conventional logistic regression in classifying stunting among school-aged children in Ethiopia. The identified predictors highlight the multifactorial and context-dependent nature of stunting. These findings support the use of ML approaches as complementary analytical tools for understanding patterns of child undernutrition, although their application for prediction should be interpreted within the limitations of cross-sectional data.

## Introduction

Childhood stunting, defined as a height-for-age Z-score (HAZ) below − 2 standard deviations of the World Health Organization (WHO) child growth standards, remains one of the most persistent forms of child undernutrition worldwide [1]. Stunting reflects chronic nutritional deprivation and recurrent infections during critical periods of growth and development, resulting in long-term consequences for physical growth, cognitive development, educational attainment, and adult productivity [2–5]. Consequently, childhood stunting represents not only a nutritional disorder but also a major constraint to human capital formation and sustainable development.

Globally, an estimated 148.1 million children under five years of age were stunted in 2022, accounting for approximately 22.3% of children in this age group [6]. Although global stunting prevalence has declined over the past two decades, progress has been uneven, with a growing concentration of the burden in low- and middle-income countries. Evidence indicates that the adverse effects of early-life stunting often persist into later childhood and adolescence, manifesting as impaired school performance, reduced physical capacity, and diminished economic productivity in adulthood [3, 4, 7].

Africa bears a disproportionate share of the global stunting burden, with more than 61 million stunted children residing on the continent, the majority of whom live in Sub-Saharan Africa [6, 8]. Unlike other regions where substantial reductions have been achieved, progress in Sub-Saharan Africa has been comparatively slow due to persistent structural challenges, including food insecurity, poverty, limited access to healthcare, inadequate sanitation, and socioeconomic inequalities [9–11]. Rapid population growth has further offset modest gains in prevalence reduction, resulting in a persistently high absolute number of affected children.

Ethiopia remains among the countries most affected by childhood stunting in Sub-Saharan Africa. National and subnational studies consistently document high levels of growth faltering and pronounced regional disparities in child nutritional outcomes [12–18]. While much of the existing evidence focuses on children under five years of age, emerging research suggests that stunting often persists into school age, where it continues to affect educational participation, cognitive development, and long-term life opportunities. Understanding stunting among school-aged children is therefore critical for capturing its broader developmental implications.

Most epidemiological studies on childhood stunting in Ethiopia have relied on cross-sectional data analyzed using conventional regression techniques, particularly logistic regression models, to examine factors associated with stunting [13, 16–19]. Although these approaches have yielded important insights into biological, maternal, household, and environmental correlates, they are limited by assumptions of linearity, restricted ability to capture complex interactions, and modest classification performance. Given that stunting arises from interdependent biological, social, and environmental processes operating over the life course, more flexible analytical frameworks are needed to better capture these relationships.

In recent years, machine learning (ML) methods have gained increasing prominence in public health research due to their ability to model nonlinear relationships, accommodate high-dimensional data, and enhance classification performance [20–22]. Several studies have applied ML techniques such as Random Forest, Support Vector Machine, and Gradient Boosting Machine models to classify childhood stunting in low-income settings, reporting improved performance compared with traditional statistical models [23–25]. In Ethiopia, recent studies employing ensemble ML algorithms have demonstrated strong performance and highlighted key socioeconomic factors associated with stunting [26].

Despite these advances, important gaps remain. Existing ML-based studies have largely emphasized predictive performance, with comparatively limited attention to model interpretability, identification of associated factors, and systematic comparison with traditional regression models treated as baseline classifiers [27]. Moreover, child-level educational and developmental indicators such as literacy skills, school participation, and school type—have received limited attention in prior ML-based stunting research, particularly among school-aged children, despite strong evidence linking nutritional status with cognitive development, educational achievement, and long-term human capital outcomes [5, 28–30]. And a relationship between height-for-age and cognitive development in school-aged children was reported by Author using multilevel analysis [31].

To address these gaps, the present study adopts an integrated analytical framework that combines logistic regression, Random Forest, Support Vector Machine, and Gradient Boosting Machine models to classify childhood stunting among school-aged children in Ethiopia using data from the Young Lives longitudinal study. Logistic regression is treated as a baseline model and systematically compared with nonlinear ML approaches using multiple performance metrics, including accuracy, sensitivity, specificity, F1-score, and area under the receiver operating characteristic curve (AUC). In addition to model performance, the study emphasizes interpretability by using odds ratios and variable importance measures to identify factors associated with stunting across child-level, household-level, educational, and environmental domains.

By explicitly incorporating educational indicators alongside nutritional and socioeconomic factors, this study provides additional insights into the nutrition education relationship and demonstrates how interpretable machine learning approaches can complement conventional epidemiological analyses. The findings aim to support evidence-based decision-making and contribute to more context-sensitive strategies for addressing childhood stunting among school-aged children in Ethiopia.

## Methods

### Study design and data source

This study employed a supervised machine learning and statistical modeling framework to identify key determinants of stunting among school-aged children and to compare the predictive performance of classical and modern classification algorithms. The analysis was based on secondary data from the Young Lives longitudinal study in Ethiopia, specifically using data from Round 5 (2016–2017) [32].

Young Lives is a large-scale, longitudinal observational study that collects detailed information on children’s health, nutrition, education, and household living conditions across multiple regions of Ethiopia. Although Young Lives follows children over time, the present analysis adopted a cross-sectional design, utilizing observations from the fifth survey round only. This approach allowed for the application of classification-based machine learning models to predict current stunting status while maintaining consistency with the available outcome and predictor variables [32].

The dataset employed in this study is publicly accessible and can be obtained by submitting a justification for the data request via the following link: https://ukdataservice.ac.uk/deposit-data/. Additional information concerning the sample, variable construction, and other relevant aspects is available in [32]. The analytical dataset comprised approximately 1,000 school-aged children, drawn from five major administrative areas (Addis Ababa, Amhara, Oromia, SNNP, and Tigray). A total of 18 predictor variables were included, capturing child-level characteristics, household socioeconomic conditions, environmental factors, school-related attributes, and parental characteristics [32].

### Outcome variable

The outcome variable was childhood stunting, defined using the World Health Organization (WHO) growth reference standards based on height-for-age z-scores (HAZ):

- Stunted: Height-for-age z-score < − 2 SD.
- Not stunted: Height-for-age z-score ≥ − 2 SD.

This binary formulation enabled the use of classification-oriented machine learning algorithms, as well as logistic regression for interpretable estimation of associations.

### Data preprocessing

#### Missing data handling

Prior to model development, the dataset was examined for missing values. Given the relatively low proportion of missing observations and to preserve the full analytical sample, simple and robust imputation strategies were employed. Continuous variables were imputed using their median values, while categorical variables were imputed using the most frequent category (mode). Median imputation was selected to reduce sensitivity to extreme values and to maintain distributional stability.

#### Data splitting

The dataset was randomly partitioned into a training set (80%) and a testing set (20%). The training set was used for model fitting and hyper parameter tuning, while the testing set served as an independent hold-out sample for final performance evaluation. This split balanced learning efficiency with unbiased assessment of predictive performance.

#### Feature encoding and scaling

Categorical predictors were encoded as factor variables suitable for classification modeling. Continuous variables, such as child weight and household size, were retained in their original scales. Feature scaling was not applied globally, as tree-based methods (Random Forest and Gradient Boosting Machine) are inherently scale-invariant, and logistic regression results were interpreted using odds ratios rather than standardized coefficients.

#### Class imbalance handling

The stunting outcome exhibited class imbalance, with a lower proportion of stunted children relative to non-stunted children. Instead of applying data-level resampling techniques, this study adopted a metric-based and algorithm-robust imbalance handling strategy. Model evaluation emphasized imbalance-sensitive and threshold-independent performance metrics, including sensitivity (recall), F1-score, and area under the receiver operating characteristic curve (AUC), rather than accuracy alone.

Additionally, ensemble learning algorithms (Random Forest and Gradient Boosting Machine), which are known to be relatively robust to class imbalance due to their tree-based structure and recursive partitioning, were prioritized. Cross-validation procedures based on ROC optimization were used during model tuning to further enhance discrimination between stunted and non-stunted children.

#### Multicollinearity assessment

To assess potential multicollinearity among predictors, Variance Inflation Factors (VIFs) were calculated for variables included in the logistic regression model. All VIF values were below commonly accepted thresholds, indicating no serious multicollinearity.

### Model development

Four predictive models were developed and compared:

1. Logistic Regression (GLM)

A multivariable logistic regression model was fitted to estimate adjusted associations between predictors and the odds of stunting. Regression coefficients were reported alongside odds ratios (ORs) and statistical significance levels.

2. Random Forest (RF)

An ensemble tree-based classifier was trained to capture nonlinear relationships and higher-order interactions among predictors. Variable importance was assessed using both Mean Decrease in Accuracy and Mean Decrease in Gini indices.

3. Support Vector Machine (SVM)

A kernel-based SVM classifier was implemented to model complex decision boundaries in the predictor space.

4. Gradient Boosting Machine (GBM)

A boosting algorithm was applied to iteratively improve classification performance by correcting errors from previous trees.

### Model validation and hyper parameter tuning

Model training and tuning were performed using repeated 10-fold cross-validation (5 repetitions) to ensure stable and unbiased performance estimates. For the Random Forest model, the optimal number of variables randomly sampled at each split (mtry) was selected by maximizing the cross-validated ROC value.

### Model evaluation

The predictive performance of all models was assessed on the same validation framework using the following metrics: Accuracy, Sensitivity (Recall), Specificity, F1-score and Area Under the ROC Curve (AUC).

Receiver Operating Characteristic (ROC) curves was generated to visually compare model discrimination. The Random Forest model’s ROC curve was additionally examined under cross-validation to confirm robustness.

### Model Interpretation

To enhance interpretability of the Random Forest model, variable importance analysis was conducted. Variables with higher Mean Decrease in Accuracy were interpreted as having stronger influence on predictive performance, while Mean Decrease in Gini reflected their contribution to node purity. Logistic regression results were interpreted using adjusted odds ratios to provide epidemiologically meaningful insights.

### Software

All analyses were conducted using R statistical software, employing standard libraries for regression modeling, machine learning, cross-validation, and performance evaluation.

### Ethical review statement

This study utilizes data from the fifth round of the Young Lives household, child, and school surveys, which are publicly accessible and archived through the UK Data Archive. Obtaining access required registration, a password application through the UK Data Service, and the signing of a confidentiality agreement. Further information on relevant research protocols is available at the following link: https://ukdataservice.ac.uk/events/ethical-and-legal-guidelines-in-data-sharing-2025-05-08/. Ethical clearance for the data collection was granted by the University of Oxford’s Central University Research Ethics Committee.

## Results

Among the 1,000 school-aged children included in the study, 32.5% were stunted, indicating a substantial burden of chronic undernutrition among school children in Ethiopia. The prevalence of stunting was slightly higher among boys (32.9%) than girls (32.0%), suggesting minimal sex-based differences in nutritional status.

**Table 1:** Descriptive characteristics of school-aged children by stunting status

Variable	Category	Stunted *n* (%)	Not stunted *n* (%)	Total *n* (%)
Sex	Male	168 (32.9)	342 (67.1)	510 (51.0)
	Female	157 (32.0)	333 (68.0)	490 (49.0)
Region	Addis Ababa	34 (22.7)	116 (77.3)	150 (15.0)
	Amhara	86 (43.0)	114 (57.0)	200 (20.0)
	Oromia	66 (33.2)	133 (66.8)	199 (19.9)
	SNNP	62 (24.8)	188 (75.2)	250 (25.0)
	Tigray	77 (38.3)	124 (61.7)	201 (20.1)
Marital status of caregiver	Permanent	241 (31.4)	526 (68.6)	767 (76.7)
	Divorced	44 (39.6)	67 (60.4)	111 (11.1)
	Single	12 (40.0)	18 (60.0)	30 (3.0)
	Widowed	28 (30.4)	64 (69.6)	92 (9.2)
School type	Public	213 (25.9)	610 (74.1)	823 (82.3)
	Private	112 (63.3)	65 (36.7)	177 (17.7)
Child work	Yes	19 (26.8)	52 (73.2)	71 (7.1)
	No	306 (32.9)	623 (67.1)	929 (92.9)
Water source	Piped into dwelling/yard	22 (22.0)	78 (78.0)	100 (10.0)
	Tube well	137 (30.0)	319 (70.0)	456 (45.6)
	Public standpipe	166 (37.4)	278 (62.6)	444 (44.4)
Toilet facility	Yes	79 (21.4)	290 (78.6)	369 (36.9)
	No	246 (39.0)	385 (61.0)	631 (63.1)
Cooking fuel	Wood	222 (32.4)	463 (67.6)	685 (68.5)
	Gas/electricity	15 (28.3)	38 (71.7)	53 (5.3)
	Cow dung	88 (33.6)	174 (66.4)	262 (26.2)
Reading ability	Cannot read	236 (43.3)	309 (56.7)	545 (54.5)
	Can read	89 (19.6)	366 (80.4)	455 (45.5)
Writing ability	Cannot write	250 (43.5)	325 (56.5)	575 (57.5)
	Can write	75 (17.6)	350 (82.4)	425 (42.5)
Residence	Urban	85 (24.2)	266 (75.8)	351 (35.1)
	Rural	240 (37.0)	409 (63.0)	649 (64.9)
Living arrangement	Both parents	219 (31.9)	468 (68.1)	687 (68.7)
	Mother only	82 (37.3)	138 (62.7)	220 (22.0)
	Father only	8 (30.8)	18 (69.2)	26 (2.6)
	Neither parent	16 (23.9)	51 (76.1)	67 (6.7)
Mother disability	Yes	38 (27.0)	103 (73.0)	141 (14.1)
	No	287 (33.4)	572 (66.6)	859 (85.9)
Mother education	No formal education	233 (35.6)	422 (64.4)	655 (65.5)
	Primary (1–8)	76 (26.9)	207 (73.1)	283 (28.3)
	Secondary+	16 (25.8)	46 (74.2)	62 (6.2)
Birth order	No older sibling	61 (30.3)	140 (69.7)	201 (20.1)
	One older sibling	55 (34.2)	106 (65.8)	161 (16.1)
	2–3 siblings	92 (34.8)	172 (65.2)	264 (26.4)
	≥ 4 siblings	117 (31.3)	257 (68.7)	374 (37.4)

Table 1 presents the distribution of stunting across socio-demographic, household, and environmental characteristics. Marked regional variation in stunting was observed. Children from Amhara (43.0%) and Tigray (38.3%) exhibited the highest proportions of stunting, whereas Addis Ababa (22.7%) had comparatively lower prevalence.

Stunting was more prevalent among rural children (37.0%) than urban children (24.2%), indicating disparities in living conditions, access to services, and food security. Higher stunting prevalence was observed among children from households where caregivers were divorced (39.6%) or single (40.0%) compared with those in permanently married households (31.4%). Similarly, children living only with their mother (37.3%) showed higher stunting prevalence than those living with both parents (31.9%).

Maternal education showed a clear gradient with stunting. Children whose mothers had no formal education had higher prevalence (35.6%) compared with those whose mothers had primary (26.9%) or secondary and above education (25.8%).

Stunting prevalence was substantially higher among children attending private schools (63.3%) compared with public schools (25.9%). Children with poor literacy skills had markedly higher stunting prevalence. Specifically, 43.3% of children who could not read and 43.5% of those who could not write were stunted, compared with 19.6% and 17.6%, respectively, among children with literacy skills.

Environmental factors also showed important patterns. Children from households without toilet facilities had higher stunting prevalence (39.0%) compared with those with sanitation access (21.4%). Similarly, higher stunting prevalence was observed among children using public standpipes (37.4%) compared with piped water (22.0%). Use of solid fuels such as wood (32.4%) and cow dung (33.6%) was also slightly higher stunting prevalence compared with cleaner energy sources.

Stunting prevalence varied modestly by birth order, with slightly higher prevalence among children with two to three older siblings (34.8%), suggesting potential intra-household resource constraints.

**Table 2:** Summary of continuous variables

Variable	Mean ± SD	Min–Max
Wealth index	0.22 ± 0.17	0.01–0.76
Household size	6.44 ± 2.16	2–16

Table 2 summarizes the continuous variables. The mean household wealth index was 0.22 (SD = 0.17), suggesting that most children came from relatively disadvantaged households. The average household size was 6.44 members (SD = 2.16), reflecting relatively large family sizes that may increase competition for household resources.

Overall, these descriptive findings indicate that childhood stunting is influenced by a combination of socioeconomic, educational, and environmental factors, supporting the application of Machine Learning to capture complex relationships among predictors.

**Table 3:** Logistic regression model results for stunting

Predictor	Estimate	Odds Ratio (OR)	Std. Error	z value	*p*-value
Intercept	−8.414	0.0002	1.461	−5.758	< 0.001
**Sex (Male)**					
Female	0.139	1.15	0.199	0.700	0.484
**Region (Addis Ababa)**					
Amhara	−0.769	0.46	0.497	−1.547	0.122
Oromia	−0.702	0.50	0.494	−1.420	0.156
SNNP	1.344	3.83	0.521	2.583	0.010
Tigray	−0.359	0.70	0.497	−0.722	0.470
**Marital Status(permanent)**					
Divorced	0.193	1.21	0.433	0.447	0.655
Single	0.435	1.54	0.589	0.737	0.461
Widowed	0.605	1.83	0.492	1.231	0.218
Household size	0.017	1.02	0.060	0.290	0.772
**School type (private)**					
Public	−1.982	0.14	0.297	−6.683	< 0.001
**Child Work (yes)**					
No	0.097	1.10	0.421	0.231	0.817
**Water source (piped into dwelling/yard/plot)**					
Tubewell in dwelling	−0.555	0.57	0.412	−1.347	0.178
Public standpipe/common tap/public well	1.175	3.24	0.485	2.423	0.015
**Toilet (yes toilet)**					
No toilet	−0.486	0.62	0.279	−1.744	0.081
**Cooking fuel (wood)**					
Gas/electricity	−0.817	0.44	0.576	−1.418	0.156
cow dung	0.153	1.17	0.248	0.618	0.536
**Child can read (yes)**					
Cannot read anything	0.622	1.86	0.172	3.626	< 0.001
**Child can write (yes)**					
Cannot write anything	0.514	1.67	0.168	3.049	0.002
**Place of residence (Urban)**					
Rural	0.686	1.99	0.372	1.844	0.065
**Living arrangement (child lives with both parents)**					
Child lives with mother but not father	−0.404	0.67	0.401	−1.009	0.313
Child lives with father but not mother	−0.948	0.39	0.575	−1.648	0.099
Child lives with neither of the biological parents	0.032	1.03	0.529	0.060	0.952
**Disabled mother(yes)**					
No	−0.399	0.67	0.304	−1.311	0.190
Wealth index	−0.878	0.42	1.444	−0.608	0.543
**Mother education** (**No formal education)**					
(1–8) primary completed	−0.020	0.98	0.261	−0.078	0.938
Secondary and above	−0.360	0.70	0.522	−0.690	0.490
**Birth order (no older sibling)**					
Child has one older sibling	−0.480	0.62	0.332	−1.443	0.149
Child has 2 to 3 older sibling	−0.349	0.71	0.300	−1.163	0.245
More than 4 sibling	−0.083	0.92	0.317	−0.263	0.792

Table 3 presents the results of the multivariable Logistic Regression analysis. After adjusting for covariates, several predictors were significantly associated with stunting. Children who could not read (OR = 1.86, *p* < 0.001) and those who could not write (OR = 1.67, *p* = 0.002) had significantly higher odds of being stunted compared with children with literacy skills. School type was significantly associated with stunting. Children attending public schools had significantly lower odds of stunting compared with those attending private schools (OR = 0.14, *p* < 0.001), consistent with the descriptive findings.

Regional disparities persisted after adjustment. Children in the SNNP region had higher odds of stunting compared with those in Addis Ababa (OR = 3.83, *p* = 0.010). Among environmental variables, children from households using public standpipes had significantly higher odds of stunting compared with those with piped water (OR = 3.24, *p* = 0.015), aligning with the descriptive results. Other variables—including sex, household size, caregiver marital status, cooking fuel, sanitation, maternal education, wealth index, living arrangement, and birth order—were not statistically significant (*p* > 0.05) after adjustment.

**Table 4:** Performance comparison of machine learning models for predicting childhood stunting

Model	Accuracy	Sensitivity	Specificity	F1-score	AUC
Logistic Regression	0.755	0.844	0.569	0.823	0.801
**Random Forest**	**0.790**	**0.896**	**0.695**	**0.852**	**0.852**
Support Vector Machine	0.770	0.889	0.523	0.839	0.818
Gradient Boosting Machine	0.770	0.807	0.569	0.826	0.832

Table 4 summarizes the predictive performance of the evaluated models in classifying childhood stunting among school-aged children based on height-for-age status. Among the models, the Random Forest algorithm demonstrated the strongest overall performance, achieving the highest accuracy (0.79), sensitivity (0.90), F1-score (0.85), and AUC (0.85). This indicates that Random Forest was particularly effective in correctly identifying stunted children while maintaining a strong balance between precision and recall.

**Fig. 1:**
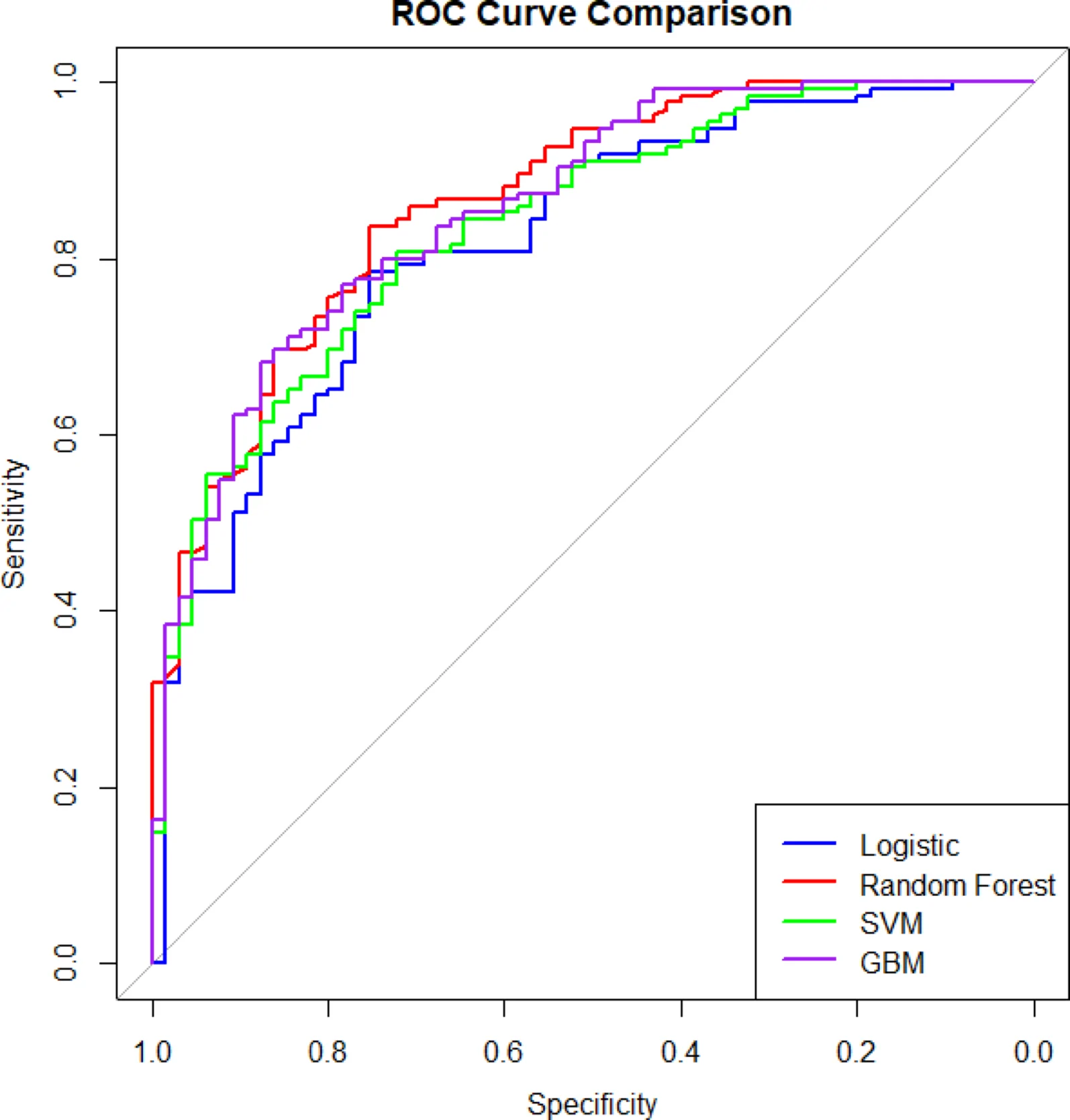
Receiver operating characteristic (ROC) curve comparison of logistic regression, random forest, support vector machine (SVM), and gradient boosting machine (GBM) models for predicting child stunting. Figure 1 compares the receiver operating characteristic (ROC) curves of the Logistic Regression, Random Forest, Support Vector Machine (SVM), and Gradient Boosting Machine (GBM) models for predicting childhood stunting among school-aged children. All models exhibit ROC curves well above the 45-degree reference line, indicating discriminatory ability substantially better than random classification

Among the evaluated models, the Random Forest model demonstrates the strongest overall discriminative performance, as evidenced by its ROC curve consistently lying above those of the other models across most threshold values. This observation aligns with its highest AUC value (0.85), reflecting superior ability to correctly distinguish between stunted and non-stunted children.

The GBM and SVM models show comparable performance, with ROC curves closely tracking one another and slightly below that of Random Forest. Both models maintain good sensitivity–specificity trade-offs, although their overall discrimination is marginally weaker than Random Forest. The logistic regression model exhibits the lowest ROC curve among the four, indicating comparatively reduced predictive power, though it still performs reasonably well.

Overall, the ROC analysis confirms that machine-learning–based models, particularly Random Forest, outperform the traditional logistic regression model in terms of classification accuracy and discriminatory ability. These findings support the use of ensemble learning approaches for improved prediction of childhood stunting in the study population.

**Table 5:** Variable importance from the random forest model based on mean decrease in accuracy and mean decrease in gini for predicting child stunting

Variable	Mean decrease accuracy	Mean decrease gini
School type	35.12	28.45
Wealth index	21.87	32.10
Child writing	19.65	18.92
Child reading	18.21	16.33
Region	16.74	19.58
Toilet facility	12.96	10.44
Place of residence	11.85	8.67
Water source	10.72	9.31
Household size	8.43	15.26
Birth order	7.15	13.02
Marital status	6.88	9.14
Living arrangement	6.22	8.75
Cooking fuel	5.91	7.83
Mother’s education	4.63	9.52
Sex	3.54	7.01
Disabled mother	3.12	5.66
Child work	2.95	4.28

Table 5 presents the variable importance measures from the Random Forest model after excluding child weight, based on Mean Decrease in Accuracy (MDA) and Mean Decrease in Gini (MDG).

The school type emerged as the most influential predictor (MDA = 35.12), indicating that it plays the largest role in classifying stunting status within the model. This suggests that differences captured by school type likely reflecting underlying socioeconomic conditions are strongly associated with child nutritional outcomes. The wealth index also showed substantial importance (MDA = 21.87; MDG = 32.10), reinforcing the critical role of household socioeconomic status in determining stunting. Child-level developmental indicators, including writing ability (MDA = 19.65) and reading ability (MDA = 18.21), remained among the top predictors, indicating a strong relationship between educational development and nutritional status.

Geographic and environmental factors, including region, toilet facility, place of residence, and water source, demonstrated moderate importance, highlighting the contribution of living conditions and sanitation to childhood stunting. Household characteristics such as household size, birth order, marital status, and living arrangement showed modest contributions, suggesting that family structure and intra-household dynamics play a secondary but relevant role.

Variables such as mother’s education, sex, disabled mother status, and child work exhibited relatively low importance, indicating limited contribution to the predictive performance of the model when stronger predictors are considered.

Overall, the Random Forest model emphasizes socioeconomic status, educational indicators, and environmental conditions as the primary determinants of stunting, providing a more policy-relevant perspective by focusing on modifiable and contextual factors rather than biological measurements.

**Fig. 2:**
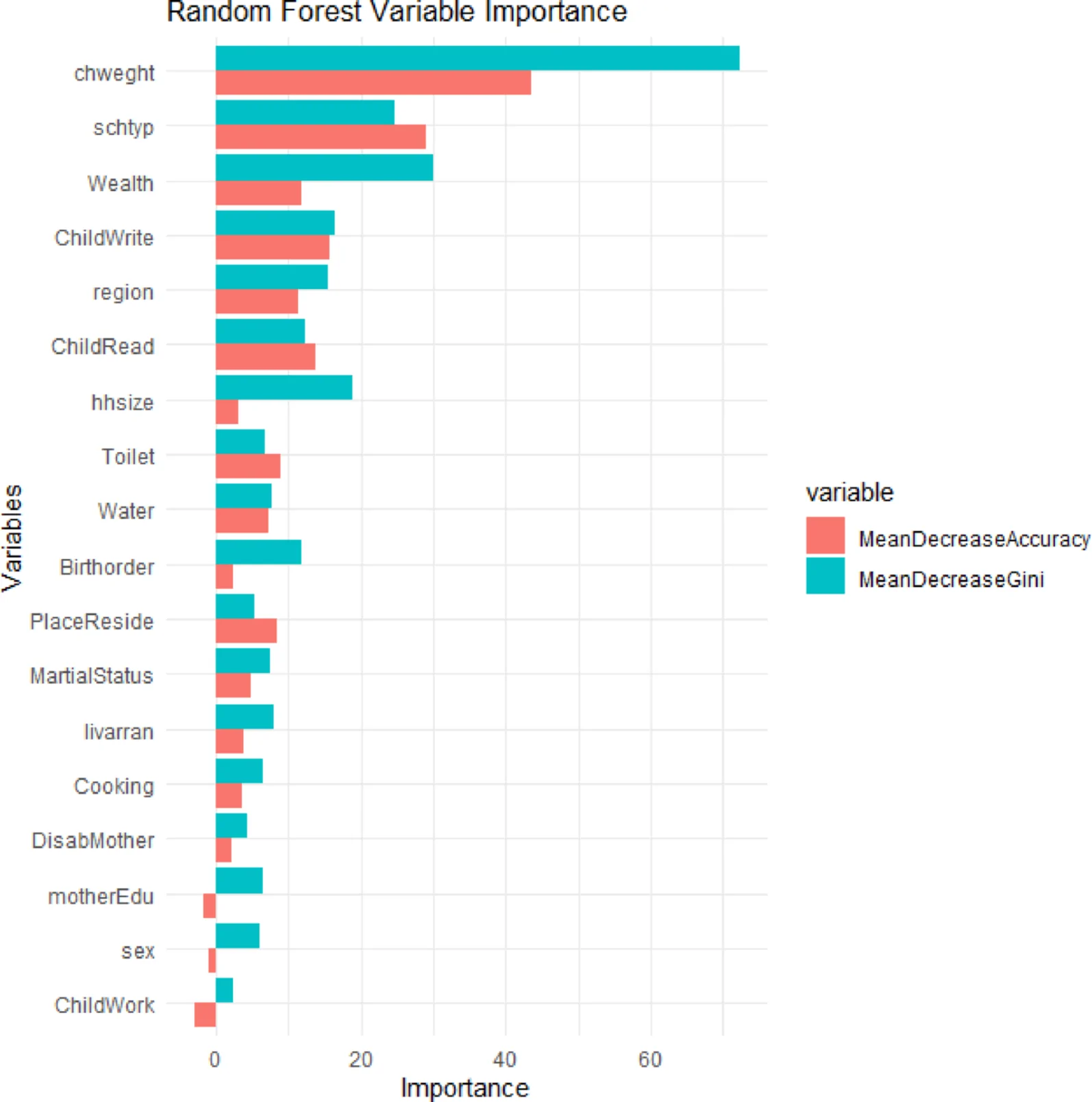
Random Forest variable importance plot based on Mean Decrease in Accuracy and Mean Decrease in Gini. Figure 2 illustrates the relative contribution of each predictor to the Random Forest model’s performance in classifying stunting status. Variables at the top of the plot have the greatest influence on prediction, while those at the bottom contribute less

The school type emerges as the most influential predictor under the Mean Decrease in Accuracy measure, indicating its dominant role in distinguishing between stunted and non-stunted children. This suggests that school type captures important underlying socioeconomic and contextual differences associated with child nutritional status. Household wealth index also shows strong importance, particularly under the Mean Decrease in Gini measure, reinforcing the critical role of socioeconomic conditions in predicting stunting. Child-level educational indicators, including writing ability and reading ability, rank among the top predictors, highlighting the close relationship between cognitive development and nutritional outcomes.

Moderate contributions are observed for region, toilet facility, place of residence, and water source, indicating that environmental and geographic factors play an important supporting role in shaping child nutrition. Household characteristics such as household size, birth order, marital status, and living arrangement contribute modestly to the model, suggesting secondary influence through intra-household dynamics. Variables including mother’s education, sex, disability status of the mother, and child work exhibit relatively low importance, indicating limited predictive contribution once stronger socioeconomic and developmental variables are accounted for.

Overall, the figure demonstrates that the Random Forest model captures complex, non-linear relationships and emphasizes socioeconomic, educational, and environmental determinants as the primary drivers of childhood stunting in the absence of dominant anthropometric predictors.

**Fig. 3:**
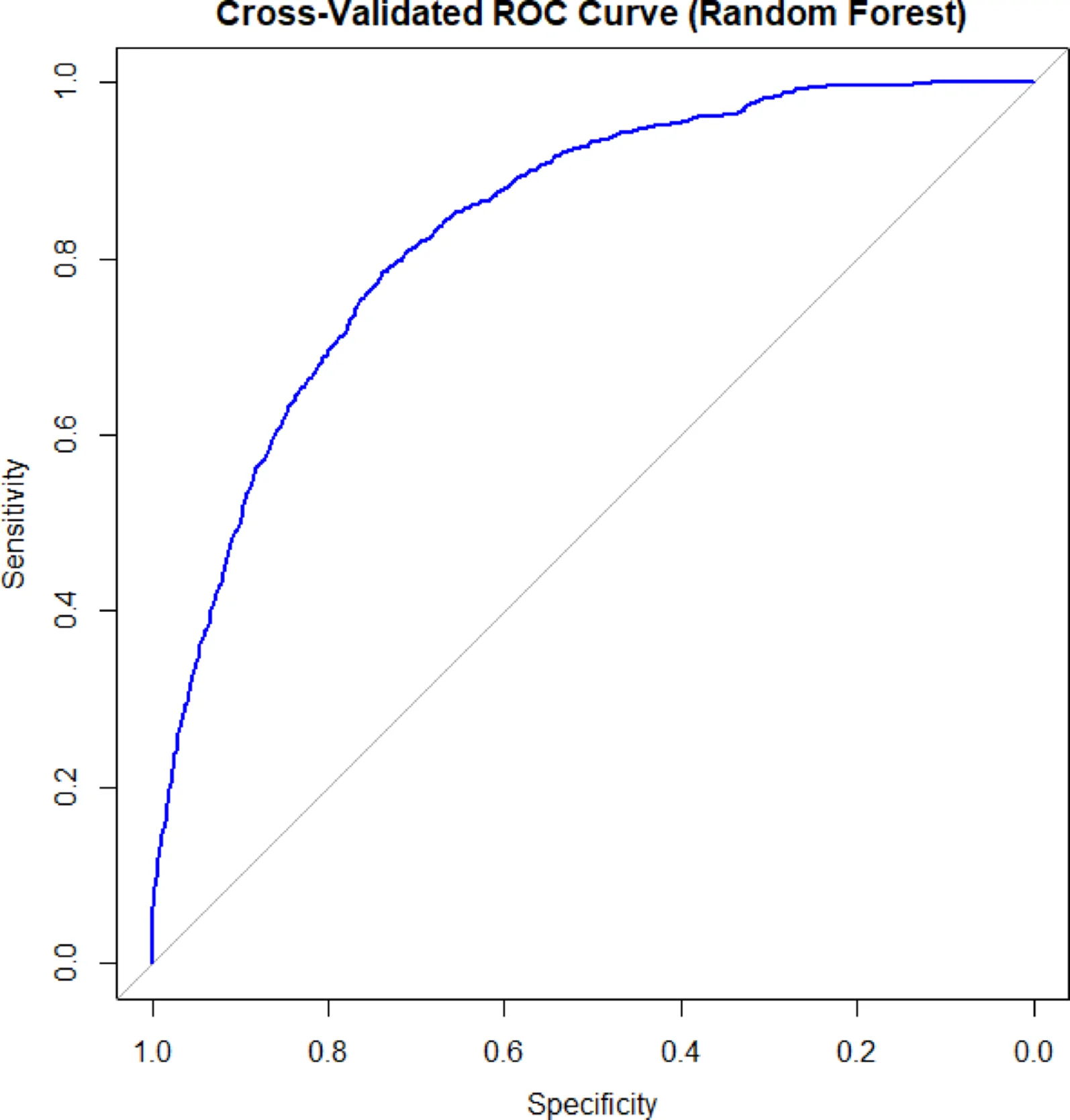
Cross-validated receiver operating characteristic (ROC) curve for the Random Forest model predicting childhood stunting. Figure 3 presents the cross-validated receiver operating characteristic (ROC) curve for the Random Forest model using 10-fold cross-validation repeated five times. The ROC curve demonstrates good discriminatory ability in distinguishing between stunted and non-stunted children. The curve lies well above the diagonal reference line, indicating performance substantially better than random classification. The corresponding cross-validated area under the curve (AUC ≈ 0.85) suggests that the model has a high probability of correctly ranking a randomly selected stunted child higher than a non-stunted child

Overall, these findings indicate that the Random Forest model provides robust and stable predictive performance across multiple resampling iterations, supporting its suitability for identifying children at risk of stunting.

## Discussion

This study applied machine learning (ML) techniques alongside multivariable logistic regression to predict childhood stunting among school-aged children in Ethiopia using school-based data. By focusing on predictive performance and associated factors, the study contributes to the growing literature on data-driven approaches to understanding childhood undernutrition beyond early childhood and outside nationally representative household surveys.

Among the evaluated models, the Random Forest algorithm demonstrated superior predictive performance, achieving the highest accuracy, sensitivity, F1-score, and area under the receiver operating characteristic curve. This finding is consistent with previous studies showing that ensemble tree-based methods perform well when applied to health and educational datasets characterized by nonlinear relationships, correlated predictors, and mixed variable types [20–22, 27]. The relatively high sensitivity of the Random Forest model indicates a strong ability to correctly identify stunted children.

Although Logistic Regression showed lower predictive performance compared with ML models, its discriminatory ability remained acceptable. This highlights the continued relevance of traditional statistical models in epidemiological research, particularly due to their interpretability and transparency. Logistic regression therefore remains a useful benchmark against which more complex ML approaches can be evaluated. The observed improvement in predictive accuracy with ML models suggests that nonlinear modeling frameworks capture additional structure in the data that may be missed by conventional linear assumptions [27] and [33].

The socioeconomic, educational, and contextual variables emerged as the most important predictors of stunting. In particular, school type, household wealth index, and child literacy indicators (reading and writing ability) showed substantial importance in the Random Forest model. These variables likely reflect broader socioeconomic and developmental conditions rather than direct causal effects. For example, limited literacy may indicate underlying disadvantages such as poor school quality, constrained household resources, or reduced cognitive stimulation, which are themselves associated with nutritional outcomes [4, 5, 28, 34, 35].

School type was identified as one of the most influential predictors, with differences observed between children attending private and public schools. This finding should not be interpreted as a direct effect of school type itself, but rather as a proxy for underlying socioeconomic and environmental differences, including household wealth, parental education, and access to better nutrition and health services. Similar patterns have been reported in previous studies linking educational context and socioeconomic status with child nutritional outcomes [7, 30, 35, 36].

Substantial regional variation in stunting risk was also observed, suggesting that geographic and contextual factors continue to play an important role among school-aged children. These differences may reflect disparities in food security, infrastructure, health service availability, and broader socioeconomic development. The persistence of regional inequalities highlights the importance of context-specific strategies to address undernutrition [9, 11, 12, 30, 37].

Household environmental factors, including water source and sanitation, demonstrated moderate importance in the Random Forest model. Their effects are likely mediated through indirect pathways such as exposure to infections and overall living conditions. The mixed patterns observed in this study are consistent with prior evidence indicating that environmental determinants interact with socioeconomic and behavioral factors in complex ways, particularly in cross-sectional analyses [36–38]. Therefore, these findings should be interpreted as indicative of associations rather than definitive relationships.

From a practical perspective, the results suggest that ML models can effectively utilize school-based data to identify patterns associated with childhood stunting. However, their application as standalone screening tools should be approached with caution. Instead, such models may serve as complementary decision-support tools alongside standard anthropometric assessments within school health and nutrition programs. Importantly, variable importance measures derived from ML models reflect predictive contribution rather than causal importance and should not be used in isolation to guide policy decisions.

**Limitations and future research**

This study has several limitations. First, the cross-sectional design limits the ability to draw causal inferences. Second, the absence of detailed dietary intake, feeding practices, and morbidity data may restrict the explanatory capacity of the models. Third, some predictor’s particularly educational variables likely function as proxies for broader socioeconomic and developmental processes, raising the possibility of reverse causality. In addition, the lack of external validation may limit the generalizability of the findings. Future research should incorporate longitudinal data, conduct validation in diverse settings, and explore the integration of additional behavioral, dietary, and environmental variables.

## Conclusions

This study demonstrates the value of integrating machine learning techniques with conventional statistical approaches to predict childhood stunting among school-aged children in Ethiopia using school-based data. The Random Forest model showed improved predictive performance compared with logistic regression, indicating its ability to capture complex and nonlinear relationships within the data.

Key associated factors identified in the analysis include school type, household wealth, literacy indicators, and region of residence, highlighting the multifactorial and context-dependent nature of stunting among school-aged children. These findings underscore the importance of considering socioeconomic, educational, and environmental contexts when examining childhood undernutrition.

While machine learning models show promise in identifying patterns within complex datasets, their role should be viewed as complementary rather than substitutive to established public health approaches. Future research should focus on longitudinal validation, improved data integration, and careful consideration of interpretability to enhance both the scientific and practical relevance of predictive models in child nutrition.

## Data Availability

The secondary data utilized for this study are available at the referenced sources.
